# *NUTM1*-Rearranged Neoplasms—A Heterogeneous Group of Primitive Tumors with Expanding Spectrum of Histology and Molecular Alterations—An Updated Review

**DOI:** 10.3390/curroncol28060381

**Published:** 2021-11-07

**Authors:** Wenyi Luo, Todd M. Stevens, Phillip Stafford, Markku Miettinen, Zoran Gatalica, Semir Vranic

**Affiliations:** 1 Department of Pathology, University of Oklahoma Health Sciences Center, Oklahoma City, OK 73104, USA; Wenyi-Luo@ouhsc.edu (W.L.); zoran-gatalica@ouhsc.edu (Z.G.); 2 Division of Anatomic Pathology, University of Alabama at Birmingham, Birmingham, AL 35294, USA; tstevens@uabmc.edu; 3 Caris Life Sciences, Phoenix, AZ 85040, USA; Phillip.Stafford@asu.edu; 4 Laboratory of Pathology, Center for Cancer Research, National Cancer Institute, Bethesda, MD 20892, USA; markku.miettinen@nih.gov; 5 College of Medicine, QU Health, Qatar University, Doha P.O. Box 2713, Qatar; 6 Biomedical and Pharmaceutical Research Unit, QU Health, Qatar University, Doha P.O. Box 2713, Qatar

**Keywords:** *NUTM1* gene, NUT protein, neoplasms, pathogenesis, therapy

## Abstract

Nuclear protein of testis (NUT), a protein product of the *NUTM1* gene (located on the long arm of chromosome 15) with highly restricted physiologic expression in post-meiotic spermatids, is the oncogenic driver of a group of emerging neoplasms when fused with genes involved in transcription regulation. Although initially identified in a group of lethal midline carcinomas in which NUT forms fusion proteins with bromodomain proteins, *NUTM1*-rearrangement has since been identified in tumors at non-midline locations, with non-bromodomain partners and with varied morphology. The histologic features of these tumors have also expanded to include sarcoma, skin adnexal tumors, and hematologic malignancies that harbor various fusion partners and are associated with markedly different clinical courses varying from benign to malignant. Most of these tumors have nondescript primitive morphology and therefore should be routinely considered in any undifferentiated neoplasm. The diagnosis is facilitated by the immunohistochemical use of the monoclonal C52 antibody, fluorescence in situ hybridization (FISH), and, recently, RNA-sequencing. The pathogenesis is believed to be altered expression of oncogenes or tumor suppressor genes by NUT-mediated genome-wide histone modification. *NUTM1*-rearranged neoplasms respond poorly to classical chemotherapy and radiation therapy. Targeted therapies such as bromodomain and extraterminal domain inhibitor (BETi) therapy are being developed. This current review provides an update on *NUTM1*-rearranged neoplasms, focusing on the correlation between basic sciences and clinical aspects.

## 1. Introduction

Ectopic expression of nuclear protein in testis (NUT), normally only expressed in the testis and ciliary ganglion [1], by gene fusions, characterizes a heterogeneous group of neoplasms [2]. What is now known as NUT carcinoma, the prototype of this group of neoplasms, was first discovered in 1991 as carcinoma with chromosomal translocation t(15:19) in two independent cases of presumed thymic primary [3,4]. A series of poorly differentiated upper respiratory tract carcinoma cases harboring the same translocation was described after that, all occurring in young patients with an aggressive clinical course [5,6,7]. The landmark discovery of the *BRD4-NUTM1* fusion gene in two independent cell lines derived from these carcinomas in 2003 provided for the genetic causality [8], which was further corroborated by functional characterization of *NUTM1* fusions such as *BRD4*-*NUTM1* [9], *BRD3*-*NUTM1* [9], and *NSD3*-*NUTM1* [10], and genomic sequencing showing a low tumor mutational burden (TMB) and lack of other oncogenic drivers [11].

The diagnosis of NUT carcinoma is affected by a nonspecific clinical presentation, histology, and immunoprofile. The initially described histologic feature of so-called “abrupt keratinization”, first described in relation to this group of tumors by Stelow et al. [12], although neither sensitive nor specific, has served as a clue to the histopathologic diagnosis. However, this clue is absent in two-thirds of the cases where NUT carcinoma is instead presented as undifferentiated carcinoma with nondescript histology [11]. Furthermore, this so-called abrupt keratinization can be seen in essentially any variant of squamous carcinoma in addition to other tumors. In addition, many non-midline cases have been reported, and some of them in soft tissue and visceral sites [13]. The correct diagnosis, therefore, relies on a strong clinical suspicion and immunohistochemical demonstration of NUT overexpression using a specific C52 NUT antibody or the presence of *NUTM1* rearrangement by fluorescence in situ hybridization or a sequencing method. It is also important to note that a subset of poroid adnexal skin tumors, including poroma and porocarcinoma, harbors *NUTM1*-rearrangement [14] and should be excluded before a diagnosis of NUT carcinoma is made on a skin-based tumor.

NUT carcinomas have been associated with varied survival. The survival analysis, facilitated by the NUT midline carcinoma registry (https://nmcregistry.org/), demonstrated dependence on fusion partners and tumor location [11]. Conventional chemoradiation therapy is of limited value for patients with NUT carcinomas [15]. The new hope lies in therapies targeting functions of fusion partners through inhibiting DNA binding by bromodomain and extraterminal domain inhibitor (BETi) [16] or downstream histone modification by NUT (histone deacetylase inhibitor (HDACi)) [17].

Recently, *NUTM1*-rearrangement has been identified in two other major types of neoplasms: hematologic malignancies and sarcomas. Up to 5% of infant acute lymphoblastic leukemia harbors *NUTM1*-rearrangement and appears to have a better prognosis [18]. The incidence and clinical course of *NUTM1*-rearranged sarcomas are presently unclear due to the small number of described cases [19,20,21,22]. However, the growing availability of comprehensive detection methods (e.g., RNA-seq) and emerging targeted therapies underline the importance of accurate diagnosis and identification of this group of emerging neoplasms. As in the oncology of other organ sites, knowledge of the specific fusion partner is often critical in selecting the appropriate therapy.

## 2. Clinical Aspects

NUT carcinoma can occur at any age. The median age at diagnosis is 23 years [11,23]. The reported earliest age of onset was in a newborn [24] and the oldest was in an 81.7-year-old [25]. No gender preference has been demonstrated [11]. It appears to afflict people worldwide [26,27]. No predisposing factor has been identified so far. Neither infectious etiology, including HPV [28] and EBV [27], nor environmental factors, including smoking [29], have been associated with NUT carcinoma. Initially discovered in the midline organs and hence named “NUT midline carcinoma” [30], NUT carcinoma has since been diagnosed in many non-midline organs [11,23]. The most common location is the lung (approximately half of the cases) followed by head and neck (one-third) [11,23]. Rare cases have been reported in the salivary glands [31,32], lacrimal sac [33], thymus, bladder [34], thyroid [35], liver/pancreas [24], brain, stomach, kidney [13], bone, and soft tissue [13]. The prevalence of NUT carcinoma is unknown. It was reported to account for 7% of poorly/undifferentiated carcinomas (PC/UCs) in children and young adults [30], up to 20% of PC/UCs in the upper aerodigestive tract (when EBV-associated tumors were excluded) [12], 12.5% of PC/UCs in the sinonasal tract [36] (with greater than 50% of sinonasal UCs in patients younger than 50 years reclassified as NUT carcinoma [37]), and 3.5% of PC/UCs in the mediastinum [38]. Only one NUT carcinoma was identified in 148 thymic epithelial tumors (including carcinomas and thymomas) in one report [39].

Other than mass lesions with local destruction and lymphadenopathy, there are no specific imaging findings [40]. Clinical symptoms and laboratory findings are nonspecific depending on the organs involved. However, it should be noted that elevated serum α-fetoprotein has been reported and may cause confusion with germ cell tumors or hepatocellular carcinoma [41]. The tumor is rapidly progressive with a 6.5-month median survival [11]. The majority of patients present with unresectable disease due to lymph node and/or organ metastasis [11]. A recent analysis of 124 patients from the NUT midline carcinoma registry demonstrated that prognosis depends on location and fusion partners [11]; thoracic tumors have the worst prognosis regardless of the translocation, with a median overall survival of only 4.4 months. They are more likely to have metastasis, larger tumor size (greater than 6 cm), and least likely to have undergone a complete surgical resection with no residual disease. The non-thoracic tumors harboring non-*BRD4*-*NUTM1* (i.e., *BRD3*-*NUTM1* and *NSD3*-*NUTM1*) have the best prognosis, with a median survival of 36.5 months. The non-thoracic tumors harboring *BRD4*-*NUTM1* have an intermediate prognosis with a median overall survival of 10 months. Other prognostic factors, including metastasis and treatment, significant in univariate analysis, were not significant in multivariate analysis. It should be noted that not all *NUTM1-*rearranged tumors are malignant. Poroma is a benign skin adnexal tumor, a subset of which harbor *NUTM1*-rearrangements and resection is curative [14]. 

There is no standard treatment for NUT carcinoma. Multimodality and intensive therapy are routinely used [11,25]. Initial surgical resection, particularly with negative resection margins [25], radiation at any time point [11], and radiation dose > 50 Gy [23] have been associated with better survival. Chemotherapy does not appear to improve survival [11]. The therapy sequence may be important for head and neck cases because patients who underwent initial surgery had the best survival, and patients who underwent initial radiation therapy had the worst prognosis [25]. The chemotherapy regimen is not standardized. Various chemotherapy drugs including Cisplatin, Carboplatin, Cyclophosphamide, Etoposide, Doxorubicin, Actinomycin D, Vinorelbine, Vinblastine, Paclitaxel, Docetaxel, 5-fluorouracil, S-1, Bleomycin, Vincristine, Ifosfamide, and Gemcitabine have been used [23]. Responses to different chemotherapy regimens were highly variable. Some patients showed a response to Ewing sarcoma regimens [42,43,44] but this therapy has not been efficacious in all patients [45]. Large-scale studies comparing the efficacy of various chemotherapy regimens are lacking.

A few small series suggested a role of programmed death-ligand 1 (PD-L1) expression in NUT carcinomas. Small-scale studies of nine cases (abstract 1191, AACR Annual Meeting 2019) and two cases [46] reported a better survival for patients with PD-L1-positive NUT carcinoma even though these patients did not receive immunotherapy. A short-duration response to anti-PD-1 therapy has been described in two of thirty-three patients with *BRD4-NUTM1* fusion, which was attributed to the high affinity of the fusion protein to MHC [23]. Other targeted therapies will be discussed below.

## 3. Histologic and Immunohistochemical Diagnosis

**Figure 1:**
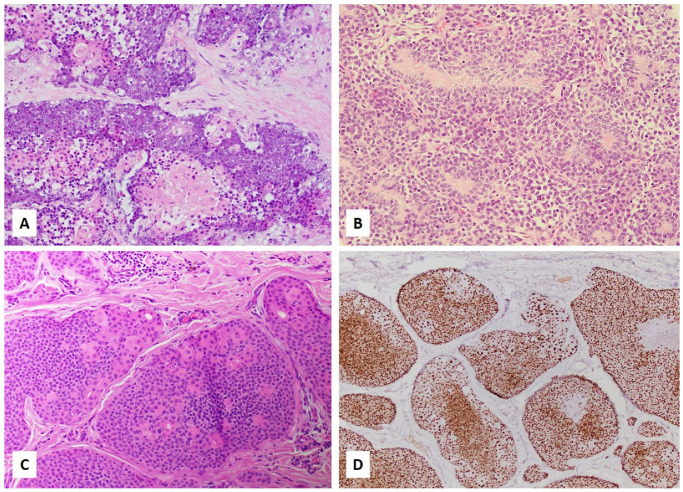
(**A**–**D**). Histology of *NUTM1*-rearranged neoplasms. (**A**) NUT carcinoma showing undifferentiated morphology with abrupt keratinization. (**B**) Small blue cell sarcoma with *MGA*-*NUTM1* fusion composed of monomorphic small blue cells forming rosettes. (**C**) Porocarcinoma with *YAP1*-*NUTM1* fusion showing features reminiscent of abrupt keratinization as seen in NUT carcinoma but having lumens and a cuticle, typical for poroid differentiation, and much more bland cytology than NUT carcinoma. (**D**) NUT immunostain in the porocarcinoma (Clone C52B1, Cell Signaling) showing diffuse and strong nuclear expression.

NUT carcinoma is composed of sheets of usually uniform tumor cells with no specific structures in most cases. Tumor cells are largely monotonous and undifferentiated with high-grade nuclei and brisk mitosis [13]. Squamous differentiation in the form of “abrupt keratinization” (Figure 1A), in other words, the abrupt appearance of small, round clusters of cells with abundant keratinized cytoplasm and shrinking of the nuclei, without a transitional area, is present in one-third of the tumors [11]. Tumor cells may grow in nests, cords, or reticular-alveolar patterns. Pseudoglandular spaces can rarely be present [13]. Focal rhabdoid tumor cells [13] or changes mimicking myoepithelial cell neoplasm such as plasmacytoid cells, or myxoid or hyalinized background have been described [13]. Biphasic tumors composed of mixed epithelial and mesenchymal components have been reported [31]. Neutrophilic infiltration has been described in some tumors of thoracic primary [47]. Therefore, a tumor with any of these features should raise suspicion for NUT carcinoma, and trigger additional confirmational studies. Due to nondescript histology in most NUT carcinomas, all poorly differentiated neoplasms, including but not limited to sinonasal undifferentiated carcinoma, HPV-associated squamous cell carcinoma, nasopharyngeal carcinoma, neuroendocrine carcinomas, adamantinoma-like Ewing sarcoma, neuroblastoma, melanoma, Ewing sarcoma, germ cell tumor, poorly differentiated carcinoma not otherwise specified, and lymphoma, should be included in the differential diagnosis. In pediatric patients, blastomas of various organs should also be included in the differential diagnosis due to their overlapping morphology with NUT carcinoma, potential multiorgan presentation, and atypical locations.

The conventional immunohistochemical diagnostic testing reveals a non-specific profile of NUT carcinoma and hence may be misleading. NUT carcinomas show frequent reactivity to epithelial markers (various cytokeratins [48] and rarely Claudin-4 [13]), SOX2 [49], and MYC [50] antibodies. However, SOX2 and MYC are not specific stains for NUT carcinoma [51,52,53,54]. Expression of the squamous markers p40 and p63 is variable and can be completely negative [55,56]. P16, a surrogate marker for HPV-associated carcinomas in specific sites (oropharyngeal and uterine cervix), is frequently positive in NUT carcinoma with no evidence of HPV (neither low-risk nor high-risk HPV) infection [28]. NUT carcinoma can show positivity for TTF1 [35,56,57] or PAX8 [56,57], potentially causing confusion with lung, thyroid, and Mullerian carcinomas, etc. NUT carcinoma frequently express neuroendocrine markers, including synaptophysin and CD56 [35], and may thus enter into the differential diagnosis with other tumors with neuroendocrine differentiation, and may also show positivity for markers suggestive of PNET including GFAP and synptophysin [13]; Ewing sarcoma markers FLI1, CD99, and cytoplasmic glycogen [45]; melanoma marker preferentially expressed antigen in melanoma (PRAME) [56]; vascular markers FLI1 and CD34 [30]; germ cell tumor markers CD30, PLAP, and SALL4 [58]; muscular markers desmin and calponin focally [55]; and hematolymphoid markers CD56 [58,59], CD30 [58], CD34, CD43 [47], CD138 [47], and CD45 [47]. Therefore, caution is advised in interpreting the results of the relatively nonspecific immunohistochemical markers. Rather, the diagnosis of a *NUTM1*-rearranged tumor is largely predicated upon the use of the widely available monoclonal C52 NUT antibody with molecular confirmation and identification of specific fusion partners in select cases. Specific NUT immunohistochemical stain should be routinely included in the initial screening marker panel of any poorly or undifferentiated neoplasm, including those beyond the midline and with unusual immunophenotypes or presentation. However, one should be cautious in interpreting the NUT immunostain because germ cell tumors can be focally NUT positive [34] without *NUTM1* gene rearrangement and rare cases are *NUTM1*-fusion positive with negative or only focal positive NUT immunostain [13,20].

The development of the highly specific monoclonal C52 antibody, which features an approximately 87% sensitivity and 100% specificity, has revolutionized the diagnosis of NUT carcinoma and *NUTM1*-rearranged tumors [34]. In general, strong nuclear reactivity in ≥50% of tumor cells was present in *NUT*-rearranged tumors in a speckled pattern. The speckles correspond to megadomains formed by chromatin and histones (discussed below). In other NUT-variant tumors except for BRD3, NSD3, and ZNF, which are within the same complex as BRD4, a homogeneous stain has been reported [21]. Focal NUT stain (as low as 10%) [26], weak stain [13], or negative stain [13,48] may occur despite increased NUTM1 mRNA levels [13], particularly in NUT-variant tumors [13]. Therefore, a strong suspicion of NUT carcinoma despite a negative C52 stain should be investigated by molecular studies. Cytoplasmic stain is considered nonspecific [34].

FISH with a probe for *NUTM1* breakpoint 15q14 was until recently considered the diagnostic gold standard [34]. However, a cryptic *NUT* breakpoint may occur and may be missed by FISH [34,60,61]. Therefore, a negative FISH in the presence of positive NUT should be investigated by alternative approaches, either by FISH using a probe spanning full-length *NUTM1* or a sequencing method, preferably RNA-seq.

Although expressions of C-MYC (8/12, 73%) and p53 (12/12, 100%) have been commonly observed, other prognostic markers have not been carefully studied. In one study, EGFR, HER2, and PD-L1 expression were observed in 2 of 7 (29%), 2 of 8 (25%), and 1 of 12 (8.3%) patients [26]. High EGFR (60%) expression has been reported in the non-squamous areas in a NUT carcinoma case [62].

## 4. Pathogenic Mechanism

*NUTM1*-rearranged neoplasms are genetically defined by a chromosomal translocation resulting in fusion of the *NUTM1* gene on chromosome 15q14 with various partners (discussed below). Although the translocation is balanced with no associated copy number changes in NUT carcinoma [63], unbalanced translocation in areas adjacent to breakpoints may occur, as demonstrated in *CIC-NUTM1* [21] and *MGA*-*NUTM1* sarcomas [20], with the former harboring occasional numerical chromosomal changes. The fusions are in-frame, and the domain of NUT responsible for binding downstream effector (the histone acetyltransferase p300) is intact [64,65].

The cell origin of NUT carcinoma is unclear. Several lines of evidence, including lack of precursor lesion, poorly differentiated morphology, differentiation potential to multiple lineages, and the expression of stem cell marker SOX2, indicate a stem cell origin. Possible neuronal derivation was suggested because NUT is normally also expressed in the adult ciliary ganglion [63]. Alternatively, they may arise from cells of different lineages upon acquiring the same oncogenic fusion as suggested by the expression of multilineage markers. Whole-genome and transcriptome sequencing revealed complex chromosomal rearrangements involving *BRD3/4–NUT*. The clustered location of breakpoints and absence of copy number change indicated that these rearrangements were likely attributable to single catastrophic events [66].

The oncogenic driver role of *NUTM1* fusion was demonstrated by functional assay showing reversal of differentiation block (evidenced by squamous differentiation of NUT cell lines) and cell cycle arrest associated with knockdown of fusion gene expression [9]. It was corroborated by a much lower overall mutation burden in NUT carcinoma cells than other cancers [67]. The mutations were largely intronic and rarely involved protein-coding genes. Among the genes involved, no canonical oncogenes or tumor suppressor genes were affected [11,66]. Similar findings of low-mutation burden and absence or rare pathogenic alterations were reported in benign poroid tumors [68,69,70].

The function of *NUTM1* fusion genes was primarily characterized in NUT carcinoma with *BRD4*-*NUT* fusion. BRD4 is a member of the bromodomain and extraterminal (BET) family, a protein family characterized by two bromodomains, an extraterminal domain, and a carboxyl-terminal domain (CTD). BRD4 is an epigenetic reader that can recognize and bind to acetylated lysines of histone 3 and 4 through its bromodomain and provide a scaffold for other factors [71,72]. BRD4, complexed with other proteins including BRD3, NSD3, and ZNF through its extraterminal domain [73], further recruits positive transcription elongation factor (pTEFb) by its CTD and activates pTEFb by displacing the negative regulators of pTEFb. The activated pTEFb can phosphorate RNA pol II and stimulate the transcription of primary response genes [74]. However, BRD4 is reciprocally regulated and hyperphosphorylated by pTEFb subunit CDK9, and the hyperphosphorylation is critical for BRD4’s function to activate transcription [75]. BRD4 hyperphosphorylation is mediated by CDK9, a subunit of TFEb. NUT is a protein expressed in post-meiotic spermatogenic cells in testis under physiological conditions and is critical for male fertility [1]. Inactivation of NUT led to male sterility with spermatogenesis arrest at the histone-removal stage [1]. Weak expression of NUT evidenced by immunohistochemical stain is also present in oocytes. Germ cell tumors, particularly dysgerminomas, had weak NUT stain, suggestive of normal NUT expression [34]. In both spermatogenic cells and NUT carcinoma cells, NUT has been shown to strongly enhance its downstream effector (the histone acetyltransferase p300) activity through direct interaction [1,65].

Unlike fusion genes in other tumors, where one of the genes only provides promoter function, both BRD4 and NUT in the fusion protein have distinct and cooperative roles in the oncogenic process. The fusion protein gains extra functionality that either protein alone does not have [65]. While retaining its functionalities in transcription, BRD4 tethers NUT to the chromosome and leads to nuclear retention of NUT protein, which is otherwise shuttling between nucleus and cytoplasm [9] and activates transcription as described above. NUT-induced epigenetic reprogramming plays a critical role in oncogenic transformation by BRD4-NUT [17]. BRD4-NUT creates an imbalance of histone acetylation across the genome, i.e., focal hyperacetylation and relative overabundance of histone deacetylase (HDAC) [17]. This is evidenced by global genomic histone hypoacetylation and reduced gene expression [17], and focal hyperacetylation status and recruitment of histone acetyltransferases CBP/p300 [65]. The hyperacetylated areas increase the binding of more BRD4 and are propagated to form megadomains through a feed-forward mechanism: initial binding of *BRD4*-*NUT* to chromosome recruit CBP/p300 and p300-mediated acetylation of adjacent nucleosomes recruit additional BRD4 and BRD4–NUT [65,76]. The fusion protein, therefore, appears to function in two opposing but nonmutually exclusive means to promote oncogenesis [77]: to increase the expression of oncogenes or anti-differentiation genes within the megadomains [77] or sequester transcription activators from p53 [65] or prodifferentiation gene *c-fo*s [78]. The function is fulfilled by an oncogenic complex composed of BRD3, NSD3, and ZNF532 (and other zinc finger proteins, ZNF592, ZNF687, and ZMYND8, collectively termed ‘Z4’ [79]), which have all been identified as NUT fusion partners in a subset of NUT carcinoma [80]. The feed-forward mechanism, therefore, covers much broader genomic coverage, which otherwise cannot be achieved by BRD4 alone. A recent study revealed the impact of mitochondrial activity on the stability of BRD4-NUT foci. Activation of the mitochondrial activity could increase the acylation/acetylation ratio of histones, especially at H4K5, and weaken BRD4-chromatin interaction [81]. Therefore, the stimulation of mitochondrial activity could potentiate the efficacy of BETi.

Many of the tumor oncogenic effects were fulfilled through the MYC and/or SOX2. Knockdown of *MYC* in NUT carcinoma cell lines induced tumor cell differentiation (which is otherwise blocked by BRD4-NUT), while forced *MYC* expression reversed tumor cell differentiation that had been induced by *BRD4*-*NUTM1* knockdown, demonstrating that *MYC* is necessary and sufficient for differentiation blockage [50]. Similar effects were observed for a stem cell marker SOX2 [49,76]. Genes encoding oncoproteins MYC and TP63 were identified within the megadomains, and their expression was upregulated [77], while p300 sequestration into the BRD4-NUT foci is the principal oncogenic mechanism leading to p53 inactivation [65]. Recently, an *MYC*-independent oncogenic mechanism has been suggested for *MXI1*-*NUTM1* fusion, which phenocopied *MYC* but did not cause *MYC* overexpression [64]. Other suggested oncogenic and regulatory mechanisms include suppression of autophagy [82], alternative splicing [83], and posttranscriptional modification [13].

Approximately two-thirds of NUT carcinoma cases harbor a *BRD4*–*NUTM1* fusion gene [68]. In the other one-third of cases, *BRD3*, *NSD3*, and *ZNFs* (*ZNF532* [58,84], *ZNF592* [79], and *ZNF618* [18]) are the partners. They are all components of the *BRD4*-*NUT* oncogenic complex [80]. We at Caris Life Sciences identified thirty-two NUT carcinoma cases in biopsies submitted for comprehensive molecular profiling using next-generation sequencing (NGS) between 2019 and 2021 (Table 1). About half of the cases had *BRD4* as the fusion partner, followed by *NSD3*, with two cases containing *BRD3*.

Many novel fusion partners of *NUTM1* were reported in other *NUTM1*-rearranged neoplasms. These include *CHRM5* [85], *BCOR1* [13], *CIC* [21], *MXD1* [13], *MGA* [19] in solid tumors and *AVEN* [60], *NAP1L4* [86], *BRD9*, *ACIN*, *CUX1*, *IKZF1*, and *SLC12A6* [18] in hematologic malignancies. Although not all of these fusion proteins have been individually studied, their pathogenic mechanisms can be postulated to be similar to *BRD4-NUTM1* since most of them are transcription regulators like *BRD4*. Probably similar to *BRD4-NUTM1*, the fusion partner of *NUTM1* tethers the fusion protein to a specific chromosomal area, and NUT initiates localized histone hyperacetylation [13,48]. Therefore, the transcription regulator portion determines what chromosomal areas are bound by fusion protein and what genes are affected [18,21,64,87]. As an example, gene profiles of the tumors with *CIC*-*NUTM1* fusion are clustered with *CIC*-rearranged sarcoma. It should be noted that confirmation of the pathogenic effects of these fusions may need to be individually verified since some of them may represent nonpathogenic stochastic passenger events [2].

Among all of the fusion partners identified so far, *SLC12A6* in leukemia and *CHRM5* in carcinoma are exceptions. Instead of being transcription regulators, *SLC12A6* and *CHRM5* are protein-coding genes. *SLC12A6* codes for a potassium-chloride co-transporter and has been demonstrated to be important in the development of the neuronal system and involved in a rare neurologic disease [88]. The lack of oncogenic drivers and increased *NUTM1* expression in leukemic cells containing *SLC12A6*-*NUTM1* fusion indicates an oncogenic role of the fusion. Similar to other *NUT* fusion proteins, nuclear retention of NUT was demonstrated in these cells [89], suggesting a similar oncogenic mechanism to other fusion proteins. *CHRM5* is a muscarinic cholinergic receptor that belongs to a larger family of G-protein-coupled receptors involved in schizophrenia [90]. The pathogenic role of *CHRM5*-*NUTM1* fusion has not been characterized yet.

Non-NUT carcinoma cell lines transfected with *BRD4*-*NUT* alone did not undergo an oncogenic transformation [63], which indicates that other factors are required for oncogenic transformation. Similarly, NUT fusion is involved in the pathogenesis of a subset of poromas and porocarcinomas with additional events likely required for a porocarcinoma to develop. Secondary events such as *KRAS*, *SETD2*, *TP53*, and *RB1* mutations are required for progression to malignancy [91]. Studying non-fusion factors is also essential in understanding the clinical heterogeneity and differential drug responses. However, these studies are still preliminary and predominantly based on next-generation sequencing (NGS) analysis. NGS has demonstrated that genes involving DNA repair are the most often mutated genes in NUT carcinoma [67]. In particular, a recurring mutation in the DNA-helicase gene *RECQL5* was detected in 75% of NUT carcinoma cell lines studied [67]. The importance of these mutations needs to be functionally verified.

## 5. Targeted Therapy

The improved understanding of pathogenic mechanisms in NUT carcinoma has led to the development of target therapies. Inhibitors of bromodomain and extraterminal (BET) proteins have been extensively studied because they are the most frequent *NUTM1* fusion partners. All the current BET inhibitors (BETi) target bromodomain (where BET proteins bind to the chromosome) and are pan-BET inhibitors [71]. Studies based on the prototype BETi JQ1 demonstrated that JQ1 replaces BRD4 from the chromosome and induces squamous differentiation and growth arrest in NUT carcinoma [16]. Twenty to thirty percent of NUT carcinoma patients treated with BETi showed partial and complete response or disease stabilization [71,92]. However, all patients relapsed. The mechanism of secondary resistance could be multifactorial. Various mechanisms have been proposed and studied in vitro on JQ1-treated NUT carcinoma cells [71,93]. Additional efforts have been made to identify multivalent BETi and explore its efficacy in non-NUT carcinoma neoplasms: a recent study demonstrated that a bivalent BET inhibitor AZD5153 (under a phase I clinical trial for refractory/relapsed solid tumor) had better tumor suppression in vivo than other monovalent BET inhibitors [26]; several BET inhibitors have entered clinical trials for patients with solid tumors and hematologic malignancies with variable success in the latter [94,95]. The limitation of BET inhibitor, in addition to its side effects, is its inability to inhibit fusions involving non-BET transcription regulators.

Selective p300 histone acetylation domain inhibitor A-485 has been identified by drug screening [96]. Treatment of NUT carcinoma cells with A-485 leads to differentiation and growth arrest, as seen for BET inhibitor [97]. C646 is another molecule that affects p300 but lacks potency or selectivity [96]. A-485 demonstrated synergistic effects with BET inhibitor [98]. NEO2734, a single agent targeting both BRD4 and p300, has shown a greater tumor suppression effect than single BET inhibitor treatment [98].

CDK9 is a subunit of pTEFb, and its inhibitor could induce robust apoptosis and DNA damage in NUT carcinoma cells. Genes affected by CDK9 inhibitor and timing of changes induced were different from those induced by BET inhibitor. Mechanistic studies demonstrated that CDK9 inhibitor utilizes a different mechanism from BET inhibitor, affecting transcriptional elongation by altering the RNA polymerase II occupancy. These findings suggest differential inhibitory pathways by CDK9 and BET inhibitors, underlying their additive effects when used simultaneously. Cross-resistance does occur due to their overlapping functions on MYC.

**Figure 2:**
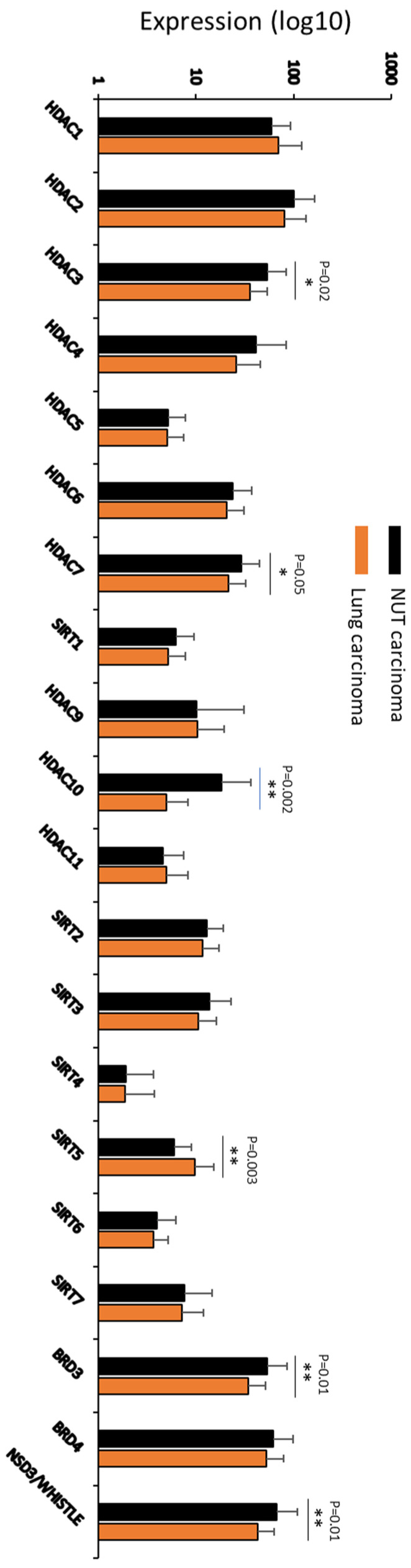
HDAC expression in NUT carcinoma. Thirty-two NUT and 22 lung carcinoma cases from biopsied patients and assayed by NGS at Caris Life Sciences between 2019 and 2021. Whole transcriptome sequencing RNA-seq (WTS) provided a measure of expression in TPM (transcripts per million molecules, as generated by Salmon). WTS used the Agilent Whole Exome SureSelect V7 bait set for RNA capture.

The epigenetic alterations in NUT carcinoma have led to therapeutic attempts with post-translational modifications of histones. Since relative overabundance of HDAC in NUT carcinoma was considered part of the pathogenic mechanism [17], HDAC inhibitors were tested. HDAC inhibitor treatment was associated with growth suppression and differentiation induction in cell lines and a mouse model. Two patients have received HDAC inhibitor treatment. A pediatric patient receiving the HDAC inhibitor vorinostat showed a marked decrease in tumor avidity for ^18^F-fluorodeoxyglucose observed by positron emission tomography [17], an early sign of tumor response. Another patient showed disease progression after the first dose of Romidepsin (a different HDAC inhibitor) [99]. A dual HDAC and PI3K Inhibitor CUDC-907 showed greater tumor suppression than BETi and was associated with increased survival in a tumor xenograft mouse model [26,100]. A case report of NUT carcinoma with increased HDAC expression in a patient with prolonged survival (4 years) raised the possible association between cellular HDAC level and prognosis [101]. We studied the HDAC expression in our NUT carcinoma cohort compared with twenty-two lung carcinoma cases identified during the same period, as shown in Figure 2. There appears to be significantly increased expression of HDAC3, HDAC7, and HDAC10 in NUT carcinoma. These findings are consistent with altered HDAC activity in NUT carcinoma. The clinical implication needs to be further determined.

Other potential therapeutic reagents include HMBA (a chemical compound that can inhibit BRD4 and p300, induce pTEFb inhibitor (HEXIM1), and inhibit BRD4 hyperphosphorylation) and CDK inhibitors such as PHA-767491 (CDC7/CDK9 inhibition), Flavopiridol (CDK1/2/4/6/7/9 inhibition), and Palbociclib (CDK4/6 inhibition) [75]. Other than HMBA and some CDK inhibitors, these molecules have not been studied in NUT carcinoma.

## 6. Other *NUTM1*-Rearranged Tumors

### 6.1. Skin Tumors

The poroid family of skin tumors includes classic poroma, hidracanthoma simplex, dermal duct tumor, poroid hidradenoma, porocarcinoma, and malignant poroid hidradenoma/poroid hidradenocarcinoma. As a whole, up to 32% of this family of skin adnexal tumors show NUT expression. However, amongst this family of poroid skin tumors, NUT expression, as assessed by immunohistochemistry, is more common in poroid hidradenoma (detected in up to 93% of poroid hidradenoma cases) and poroid hidradenocarcinoma (detected in up to 80% of poroid hidradenocarcinoma cases). In the order of 17% and 11% of classic poroma and porocarcinoma cases, respectively, show NUT expression. In addition, amongst all other subtypes of cutaneous adnexal tumors tested, NUT expression is only found in a subset of poroid skin tumors [91]. Figure 1C,D show a porocarcinoma that harbored a *YAP1-NUTM1* fusion and its corresponding NUT immunostain.

The majority of fusion transcripts in poroid tumors detected were *YAP-NUTM1*, *YAP-MAML2*, and/or *MAML2-YAP* fusion [14,69,91]. Rare tumors harbor *WWTR1-NUTM1* [14] and *EMC7-NUTM1* [91] fusions. The simultaneous presence of these transcripts was common except *WWTR1-NUT1*, which was mutually exclusive with *YAP1-NUTM1* [14] (*YAP-NUTM1* and *EMC7-NUTM1* are not mutually exclusive and have been detected in the same tumor [91]). Fusions are all in-frame with preserved transcriptional enhancer factor domain TEA DNA binding domain (TEAD)-binding and p-300-binding domain [14,69]. In vitro experiments demonstrated that these fusion genes activated TEAD transcription factors and promoted anchorage-independent growth of epithelial cells [14].

Pathologists should not be quick to jump to a diagnosis of NUT carcinoma for a skin tumor featuring abrupt keratinization, immunohistochemical evidence of squamous differentiation (positive cytokeratin 5/6 and/or p63), and NUT expression, as the poroid family of skin tumors may share these features. Features of ductal and cuticular differentiation as well as low-grade, bland nuclei, features associated with the poroid family of tumors, should be sought (with the aid of cytokeratin 7 stain when subtle). RNA fusion analysis to identify the fusion partner may be considered for challenging cases. The distinction between *NUTM1*-rearranged poroid tumor and NUT carcinoma has a profound impact on a patient’s treatment and prognosis. Even the malignant poroid tumor tends to have an indolent clinical course, and complete resection may be curative [70].

### 6.2. Sarcomas

*MXD* and *MGA* are members of the MAX dimerization (*MAD*) gene family, including proteins that engage MAX as a cofactor for DNA binding and controlling gene expression [64]. Heterodimerization of MXD4 or MGA with MAX results in transcriptional repression of E-box target DNA sequences [48]. Transcriptome analysis showed paradoxical enrichment of MYC target genes in the *MXD2/MXI1-NUTM1* tumor and promoted cell proliferation and anchorage-independent growth despite the lack of MYC expression, suggesting an “MYC-like” function [64].

*MGA*-*NUTM1* sarcoma has been described in soft tissue (thigh and foot) [19,20], dura [20], and thorax (lung and chest wall/pleura) [48]. These tumors are composed of the dense monomorphic spindle to slightly epithelioid cells and stroma that is myxoid [48], collagenous [20], or associated with matrix deposition resembling osteoid [20,48]. These tumors showed variable NUT (mostly diffuse and strong with one case being multifocal and weak [20]) and CD99 expression [19,20]. One tumor demonstrated aggressive behavior despite low histopathologic grade [19]. Chemotherapy combined with local control by surgery and radiation was associated with disease-free survival of 132 months in one case [20]. *MGA* and *NUTM1* fusion is likely generated by inversion since they are located 7.4 Mb apart on the same chromosome (chromosome 15) in opposite orientations [48]. Figure 1B shows a small round cell sarcoma harboring *MGA*-*NUTM1* fusion. The tumor is composed of monomorphic small blue cells with rosette formation.

One ovarian [68] and five colon [22] *MXD4-MUTM1* fusion sarcoma cases have been reported. The tumors in the ovary and colon showed similar morphology with small round cells and spindle cells. Fibrosarcomatous (herringbone) pattern, epithelioid/rhabdoid pattern with cells growing in sheets, and hyalinized/nested pattern are variably present. These tumors showed variable weak CD34, smooth muscle actin, CD99, ERG, and synaptophysin expression. *MXD2/MX11*-*NUTM1* fusion has been reported in a sarcoma centered on the stomach and involved the distal esophagus [64]. The tumor was composed of nests, sheets, and strands of discohesive, monotonous cells associated with a small amount of myxoid stroma. The tumor was negative for cytokeratins, p40, SOX2, and MYC and positive for NUT, FLI1, and CD99. These tumors showed aggressive behavior and were universally lethal. Dr. Andrew Folpe has recently coined the term “*NUTM1-*rearranged colorectal sarcoma” to codify this group of tumors in the colon [22].

An intramuscular *BCOL1-NUTM1* fusion sarcoma has been described [13]. The tumor had spindle and epithelioid morphology with focal rosette formation and showed no reactivity to NUT antibody but significant *NUTM1* mRNA expression, suggesting posttranscriptional modification. The patient suffered from soft tissue, lung, and lymph node metastasis and died of disease at 48 months.

*CIC-NUTM1* fusion has been identified in round cell tumors of the pediatric population and young adults [21,87,102,103]. These tumors displayed morphologic features similar to bona fide *CIC-DUX4* sarcomas, namely lobulation, focal spindling, myxoid changes, distinct nucleoli, and positive ETV4 and WT1 immunohistochemical stains [21]. Rhabdoid morphology can be focally present [21,103,104]. Gene profiles of these tumors show clustering with *CIC*-rearranged sarcoma rather than NUT carcinoma [21,87]. A case of head and neck tumor harboring *CIC*-*NUTM1* fusion in an adult with features of myoepithelial carcinoma was also reported [104]. The tumor had no expression of ETV4 and WT1, markers typically positive in *CIC*-rearranged sarcoma and which may be useful in this differential diagnosis. Therefore, NUT carcinoma was favored over *CIC*-rearranged sarcoma [104].

### 6.3. Brain Tumors

The *BRD4-NUTM1* NUT carcinoma in the parietal lobe of a 3-year-old patient had a reticular-alveolar morphology and showed focal GFAP and synaptophysin positivity mimicking neuroepithelial neoplasm [13]. The patient died of disease 12 months after surgery and chemotherapy. Two other pediatric primitive neuroectodermal tumors (PNETs) containing *CIC*-*NUTM1* were identified in an international study aimed at PNET molecular classification [102]. However, no clinical information was described. The *ATXN1-NUTM1* tumor in the frontal lobe of a 21-year-old young adult showed primitive spindle cells in chondromyxoid morphology with strong GFAP positivity [105]. The patient was disease-free 16 months after surgery and additional therapy. The *PARD3B-NUTM1* tumor discovered in a 29-year-old female showed primitive spindle cells in a myxoid to fibrillary background and foci of small epithelioid cells and had an aggressive clinical course [59].

### 6.4. Hematologic Neoplasms

*NUTM1* rearrangement has been identified in both acute and chronic hematologic malignancies.

*NUTM1* rearrangement was more common in B cell precursor acute lymphoblastic (B-ALL) of infants (3–5%) than in children (0.4–0.9%). Up to 21.7% of *KMT2A*-wildtype B-ALL in infants enrolled in interfant studies (a large international consortium on infant acute lymphoblastic leukemia) harbored *NUTM1*-rearrangement. Ten fusion partners (*ACIN1*, *CUX1*, *ZNF618*, *SLC12A6*, *CHD4*, *BRD9*, *AFF1*, *ATAD5*, *RUNX1,* and *IKZF1*) have been identified in infant and pediatric forms of B-ALL [18]. *ACIN1* (44%), *BRD9* (26%), and *CUX1* (15%) were most common in infants, and *CUX1* (28%), *ZNF618* (28%), and *ACIN1* (22%) were most common in children. *NUTM1*-rearrangement is mutually exclusive with other sentinel leukemia-driving aberrations in *IKZF1*, *PAX5*, *ETV6*, or *CDKN2A/B*. *NUTM1*-rearranged B-ALL was associated with *BMI1* (a proto-oncogene, which enhances the self-renewal of hematopoietic stem cells and can convert *BCR-ABL1*-positive progenitor cells to acute lymphoblastic leukemia) upregulation [89]. Some fusion partners, including *ACIN1*, *CUX1*, *BRD9*, and *AFF1*, were associated with *HOXA* gene cluster upregulation [89], which has been associated with sensitivity to inhibitors of KMT2A-Menin complex [106]. *NUTM1*-rearranged B-ALL has a favorable outcome. In the study by Judith M. Boer et al., the 4-year overall survival was 100% in *NUTM1*-rearranged cases versus 74.0% in *NUTM1*-wildtype/*KMT2A*-wildtype cases while 4-year relapse survival was 92.1% [18].

Two *NUTM1*-rearranged myeloid neoplasm cases were reported. The *NAP1L4*-*NUTM1* fusion gene was identified in a case of myeloid neoplasm with eosinophilia and *PDGFRA* rearrangement with negative *ETV6*-*PDGFRβ*, *BCR-ABL1*, and *PCM1-JAK2* [86]. The treatment led to the vanishment of *FIP1L1*-*PDGFRA* and *NAP1L4*-*NUTM1*, suggesting a potential interaction between these two fusion genes/proteins. The patient had an atypical blast-like presentation and a good response to imatinib mesylate therapy. The *AVEN*-*NUTM1* fusion gene was identified in a case of acute myeloid leukemia with monocytic differentiation [60]. The patient had multiple recurrences even after allogenic hematopoietic stem cell transplantation. The fusion was cryptic and created by *AVEN* inversion, which can be easily missed by routine karyotype analysis. However, the pathogenic role of the fusion protein is unclear due to the simultaneous detection of *IDH1* and *RUNX1* mutations.

It is worth noting that NUT carcinoma may rarely present with a “leukemic phase” when peripheral blood and bone marrow are involved [107]. A misdiagnosis of CD34-positive acute leukemia should be avoided.

## 7. Conclusions

The year 2021 marks the 30th anniversary of the first description of NUT carcinoma. With pioneers in the field, such as Dr. Christopher French, significant progress has been made in the understanding of the pathogenesis, molecular alterations, and clinicopathologic features of this group of neoplasms. This review serves as a comprehensive summary of these discoveries, focusing on molecular, pathologic, and clinical correlations. We also presented new cases from 2019 to now emerging from the more frequent use of NGS in diagnostic pathology.

There are still many unanswered questions. These include but are not limited to the incidence, precise pathologic classification, and clinical behavior of *NUTM1*-rearranged neoplasms harboring different fusion partners, mechanisms of oncogenic transformation, the functionality of variant translocations, and the impact of the genomic background. Equally important is the screening of targeted therapies and characterization of drug resistance mechanisms.

## Figures and Tables

**Table 1:** NUT carcinoma cases identified by next-generation sequencing at Caris Life Sciences between 2019 and 2021.

Partner	Primary Tumor Site	Histology	Specimen Site
BRD4	Skin, NOS	NUT carcinoma	Liver
BRD4	Upper lobe, lung	Adenocarcinoma, metastatic, NOS	Arm, NOS
BRD4	Lower lobe, lung	Squamous cell carcinoma, NOS	Mediastinum, NOS
NSD3	Lung, NOS	Basaloid carcinoma	Inguinal region, NOS
BRD4	Paranasal sinus	Carcinoma, NOS, midline carcinoma of children and young adults with NUT rearrangement	Eyelid
NSD3	Lung, NOS	Squamous cell carcinoma, NOS	Lung, NOS
NSD3	Upper lobe, lung	Squamous cell carcinoma, NOS	Pleura, NOS
BRD4	Lower lobe, lung	NUT midline carcinoma	Mesentery
BRD4	Upper lobe, lung	Squamous cell carcinoma, NOS	Upper lobe, lung
NSD3	Lower lobe, lung	NUT midline carcinoma	Mediastinal lymph node
BRD4	Nasal cavity	NUT carcinoma	Nasal cavity
BRD4	Nasopharynx, NOS	Squamous cell carcinoma, NOS	Nasal cavity
BRD4	Upper lobe, lung	Squamous cell carcinoma, metastatic, NOS	Mediastinal lymph node
BRD4	Frontal sinus	NUT midline carcinoma	Frontal sinus
BRD4	Upper lobe, lung	Carcinoma, NOS	Pleura, NOS
NSD3	Lower lobe, lung	Non-small cell carcinoma	Lower lobe, lung
BRD4	Upper lobe, lung	NUT midline carcinoma	Upper lobe, lung
NSD3	Maxillary sinus	NUT carcinoma	Lymph node, NOS
NSD3	Lung, NOS	Carcinoma, undifferentiated, NOS	Lung, NOS
NSD3	Unknown primary site	NUT carcinoma	Mediastinal lymph node
BRD4	Mediastinum, NOS	Neoplasm, malignant	Mediastinum, NOS
NSD3	Lung, NOS	NUT midline carcinoma	Supraclavicular lymph node
BRD3	Lower lobe, lung	NUT carcinoma	Lower lobe, lung
BRD4	Ethmoid sinus	Malignancy, NUT carcinoma	Eye, NOS, Orbit, NOS
BRD4	Upper lobe, lung	NUT carcinoma	Subclavicular lymph node
BRD3	Upper lobe, lung	NUT carcinoma	Upper lobe, lung
NSD3	Thyroid, NOS	Carcinoma, metastatic, NOS	Pretracheal lymph node
BRD4	Upper lobe, lung	NUT midline carcinoma	Upper lobe, lung
BRD4	Nasal cavity	NUT carcinoma	Skull, NOS
BRD4	Upper lobe, lung	Squamous cell carcinoma, NOS	Upper lobe, lung
NSD3	Lung, NOS	Squamous cell carcinoma, NOS	Main bronchus
NSD3	Thyroid, NOS	Carcinoma, NOS	Thyroid gland

NOS—Not otherwise specified.

## Data Availability

Available from the corresponding author at a reasonable request.
